# Dynamic Regulation of Ferroptosis in a Neonatal Rat Model of Postnatal Hypoxia-Induced Acute Kidney Injury

**DOI:** 10.3390/antiox15050582

**Published:** 2026-05-04

**Authors:** Tzu-Hao Liu, Bo-Hau Chen, Guan-Hong Lin, Hsin-Hung Chen, Hsiang-Chin Chiu, Chih-Chieh Yang, Yi-Ting Chu, Ching-Ming Lin, Wen-Hsien Lu

**Affiliations:** 1 Department of Pediatrics, Zuoying Armed Forces General Hospital, Kaohsiung 813204, Taiwan; 2 Department of Pediatrics, Taoyuan Armed Forces General Hospital, Taoyuan 325208, Taiwan; 3 Department of Pediatrics, Kaohsiung Veterans General Hospital, Kaohsiung 813414, Taiwan; 4 Department of Medical Education and Research, Kaohsiung Veterans General Hospital, Kaohsiung 813414, Taiwan; 5 Department of Pediatrics, Pingtung Veterans General Hospital, Pingtung 900053, Taiwan; 6 Department of Pediatrics, Kaohsiung Armed Forces General Hospital, Kaohsiung 802301, Taiwan; 7 Department of Health-Business Administration, Fooyin University, Kaohsiung 831301, Taiwan; 8 Institute of Biomedical Sciences, National Sun Yat-sen University, Kaohsiung 804201, Taiwan

**Keywords:** acute kidney injury, ferroptosis, fibrosis, postnatal hypoxia, reoxygenation

## Abstract

Hypoxia during the postnatal period represents a significant risk factor for renal injury in neonates, yet the molecular mechanisms linking oxygen deprivation to kidney damage remain unclear. In this study, we explored whether ferroptosis serves as a key mediator in hypoxia-induced acute kidney injury (AKI). Using a neonatal rat model, we demonstrated that hypoxic exposure disrupts iron homeostasis, leading to iron accumulation and excessive lipid peroxidation in renal tissues. These alterations were associated with marked tubular injury, glomerular damage, and progressive fibrotic remodeling. Importantly, reoxygenation attenuated ferroptosis-related signaling pathways and improved renal structural and functional outcomes, although incomplete recovery was observed in fibrotic changes. Our findings suggest that ferroptosis is not only involved in the initiation of hypoxia-induced renal injury but may also contribute to its progression toward chronic kidney pathology. Targeting ferroptosis and iron metabolism may therefore represent a promising strategy for preventing or treating neonatal hypoxia-related AKI.

## 1. Introduction

Several pathophysiological conditions, including congenital cardiopulmonary disorders, respiratory distress syndrome, nuchal cord, anemia, or neonatal shock caused by sepsis or allergic reactions, can cause postnatal hypoxia in newborns during the postpartum period [1,2,3]. When these cardiorespiratory pathophysiological abnormalities occur, neonates may develop persistently low arterial oxygen saturation (SaO_2_) after birth. Prolonged inadequate oxygenation may interfere with growth and development over a long period and may also induce systemic multi-organ injury [3,4].

The kidneys are highly sensitive to changes in blood flow and oxygen perfusion. When neonates remain in a state of hypoxia, renal tubular epithelial cells in the cortex may undergo injury and activation of cell death pathways due to the accumulation of reactive oxygen species (ROS), ultimately leading to acute kidney injury (AKI) [5]. Epidemiological studies have demonstrated that AKI has a high incidence among neonates suffering from asphyxia and is associated with increased mortality in neonatal intensive care units [5,6]. Clinically, the incidence of AKI is strongly correlated with the severity of neonatal asphyxia [5,6]. Notably, among infants who develop AKI, the need for mechanical ventilation has been reported to be significantly associated with a markedly increased risk of mortality [7]. In addition, our previous study revealed that renal injury occurred in rat pups exposed to hypoxic conditions from birth for seven days [8]. Although previous studies have demonstrated a strong association between perinatal hypoxia and AKI, the underlying mechanisms and effective therapeutic strategies remain poorly understood.

Ferroptosis is a form of non-apoptotic cell death characterized by iron accumulation and lipid peroxidation, as first described by Dixon et al. [9]. Under pathological stress conditions, dysregulation of cellular iron homeostasis leads to the accumulation of redox-active iron, which contributes to cellular damage. Ferroptosis is generally driven by three major processes, including iron metabolism dysregulation, impairment of the glutathione-dependent antioxidant system, and lipid peroxidation. Intracellular iron is utilized for the synthesis of iron-containing proteins, while excess iron is either stored in ferritin or exported via ferroportin to maintain iron homeostasis. During ferritin storage, ferrous iron (Fe^2+^) is oxidized to ferric iron (Fe^3+^) by the ferroxidase activity of the ferritin heavy chain (FtH), followed by mineralization into ferrihydrite (Fe_2_O_3_·H_2_O) within the ferritin core mediated by the ferritin light chain (FtL) [10]. Under conditions of iron overload or oxidative stress, ferritin-bound ferric iron can be reduced to ferrous iron and released into the cytoplasm, contributing to the expansion of the labile iron pool and promoting ferroptosis. These ferrous ions catalyze the formation of reactive oxygen species through Fenton reactions and also act as cofactors for arachidonatelipoxygenases (ALOXs), leading to the generation of lipid peroxides (LP-OOH) from polyunsaturated fatty acids (PUFAs) such as arachidonic acid [11]. The accumulation of lipid peroxides overwhelms the glutathione-dependent antioxidant defense system, particularly glutathione peroxidase 4 (GPX4), resulting in oxidative damage to cellular membranes. Furthermore, lipid peroxides can propagate chain reactions that disrupt phospholipid structures, impair membrane integrity, and ultimately lead to cell death [12,13,14].

Although several studies have identified ferroptosis as a critical cell death pathway in hypoxic injury, investigations into the relationship between ferroptosis and postnatal hypoxia have primarily focused on cerebral or neurological damage [15,16,17]. Previous studies have also demonstrated that ferroptosis is involved in AKI induced by various risk factors, including ischemia/reperfusion injury, sepsis, cisplatin exposure, and other triggers [18,19]. However, it remains unclear whether ferroptosis contributes to the pathophysiological mechanisms underlying postnatal hypoxia-induced AKI.

Our previous study demonstrated that newborn rats developed pathological renal injury and decreased renal function after exposure to a hypoxic environment for seven days from birth [8]. Based on these findings and previously published reports, we hypothesized that ferroptosis may be involved in postnatal hypoxia–induced AKI. To investigate the relationship between ferroptosis and hypoxic renal injury, as well as the dynamic changes in kidney injury following medical correction of arterial oxygen saturation (SaO_2_), we employed an established rodent model mimicking postnatal hypoxia in newborns. Using this model, we compared the effects of ferroptosis following 7-day and 14-day hypoxic exposure (13% oxygen) [8]. Furthermore, to simulate clinical conditions in which SaO_2_ is restored, animals exposed to hypoxia for seven days were subsequently returned to normoxic conditions (21% oxygen) for an additional seven days, allowing further evaluation of ferroptosis-related pathways and AKI pathophysiology.

## 2. Materials and Methods

### 2.1. Experimental Animals

Pregnant female Sprague-Dawley rats (RRID: RGD_70508) were purchased from BioLASCO Ltd. (Yilan County, Taiwan) and housed at the Experimental Animal Center of Kaohsiung Veterans General Hospital (KSVGH, Kaohsiung City, Taiwan). Animals were provided with food and water ad libitum and maintained under controlled environmental conditions with a 12 h light/12 h dark cycle in accordance with the guidelines of the Institutional Animal Care and Use Committee (IACUC) of KSVGH and the Ministry of Agriculture, Taiwan.

### 2.2. Animal Model Design

Within 12 h after birth (postnatal day 0, P0), neonatal rats were housed with their dams in a hypoxia chamber. Nitrogen and ambient air were supplied to maintain the oxygen concentration at 13% and the carbon dioxide concentration below 1200 ppm to prevent respiratory acidosis in both dams and pups. Normoxia control pups were housed under normal atmospheric oxygen conditions. For the reoxygenation group, pups were transferred from the hypoxia chamber to normoxic cages at postnatal day 7 (P7) and maintained under normoxic conditions until postnatal day 14 (P14). All pups were anesthetized with isoflurane and euthanized by decapitation at P7 or P14 for subsequent tissue and blood collection. All pups were divided into five groups: those maintained under normoxic conditions for 7 days (N7) or 14 days (N14), those exposed to hypoxic conditions for 7 days (H7) or 14 days (H14), and those exposed to hypoxic conditions for 7 days followed by a return to normoxic conditions for an additional 7 days (H7N7).

### 2.3. Blood Gas and Biochemical Analysis

Fresh blood samples collected from decapitated pups were transferred into 1 mL heparin-coated syringes to prevent coagulation. Samples were immediately analyzed in the pediatric laboratory at KSVGH for blood gas parameters and biochemical indices. Blood oxygen and carbon dioxide concentrations, as well as electrolyte levels, were measured using a blood gas analyzer.

### 2.4. Semi-Quantification of Pathomorphological Injuries

Paraffin-embedded kidney sections were stained with periodic acid-Schiff (PAS) staining (BioTnA, Kaohsiung, Taiwan; TASS02) to evaluate renal pathomorphological injury. Based on the criteria described below, tubular injury score (TSI) and glomerulosclerosis index (GSI) were used to assess and distinguish the severity of pathological changes in renal tubules and glomeruli within the renal cortex [20].

TSI was determined according to the area fraction of tubulointerstitial injury in each cortical field observed at 200× magnification. Pathological features included tubular dilation, tubular atrophy, loss of the brush border, hyaline cast formation, thyroidization, interstitial inflammation, and fibrosis. The severity of injury was graded on a six-point scale as follows: (1) Grade 0: no detectable pathological injury; (2) Grade 1: pathological injury involving <10% of the field area; (3) Grade 2: pathological injury involving 10–25% of the field area; (4) Grade 3: pathological injury involving 25–50% of the field area; (5) Grade 4: pathological injury involving 50–75% of the field area; and (6) Grade 5: pathological injury involving >75% of the field area. The final TSI for each sample was calculated as the mean score of all examined cortical fields.

GSI was evaluated based on the area fraction of glomerular abnormalities observed at 400× magnification. Morphological criteria included basement membrane thickening, mesangial extracellular matrix expansion (PAS-positive area), lobulation or atrophy of the capillary tuft, and crescent formation [20]. Glomerular injury severity was graded on a five-point scale as follows: (1) Grade 0: no detectable glomerular abnormality; (2) Grade 1: abnormalities involving <25% of the glomerular area; (3) Grade 2: abnormalities involving 25–50% of the glomerular area; (4) Grade 3: abnormalities involving 50–75% of the glomerular area; and (5) Grade 4: abnormalities involving >75% of the glomerular area. The final GSI for each sample was calculated as the mean score of all evaluated glomeruli within the tissue section.

### 2.5. Quantification of Renal Fibrosis

Paraffin-embedded kidney sections were post-fixed in Bouin’s solution (Sigma-Aldrich, St. Louis, MO, USA; HT10132) and stained with Picro-Sirius Red (PSR) (Abcam, Cambridge, UK; ab245887) to detect collagen deposition. Images were captured at 400× magnification, and the fraction of PSR-positive area in the renal cortex was quantified using ImageJ software (version 1.48).

### 2.6. Detection of Iron Ions

Free ferric ions in kidney sections were detected using a Prussian blue staining kit (TASS15; BioTnA; Kaohsiung, Taiwan) according to the manufacturer’s instructions. Prussian blue precipitates formed by the reaction of potassium ferrocyanide with ferric ions were enhanced using 3,3′-diaminobenzidine (DAB) [21]. Images were acquired at 200× magnification for the cortex and 400× magnification for glomeruli, and iron accumulation was quantified as the DAB-positive area fraction using ImageJ.

### 2.7. Immunohistochemistry Staining (IHC)

Paraffin-embedded kidney sections underwent heat-induced antigen retrieval using 0.01 M sodium citrate buffer (pH 6.0) or a commercial EDTA-based retrieval solution. Immunohistochemical staining was performed using the Novolink^TM^ Polymer Detection System (Leica Biosystems Nussloch GmbH, Nussloch, Germany; RE7280-K), followed by incubation with primary antibodies. Immunoreactivity was visualized using DAB. Images were captured at 200× or 400× magnification for the renal cortex and 400× magnification for glomeruli. Protein expression levels were quantified by measuring mean gray density using ImageJ. Primary antibodies included rabbit anti-mouse Alox-12 (1:200; ABclonal, Woburn, MA, USA; A14703), ferritin heavy chain (1:3000; ABclonal; A1144), ferritin light chain (1:100; ABclonal; A21758), FGF-23 (1:200; Bioss, Woburn, MA, USA; bs-5768R), HIF-1α (1:500; Proteintech, Rosemont, IL, USA; A23150), Klotho (1:200; Proteintech; 28100-1-AP), NGAL (1:1000; Abcam; ab63929) and nephrin (1:200; A23150; ABclonal).

### 2.8. Statistics

All histological images were captured using a BX51P polarizing microscope (Olympus, Tokyo, Japan) and analyzed using ImageJ software (National Institutes of Health, Bethesda, MD, USA). Data normality was assessed using the Shapiro–Wilk test. For normally distributed data, parametric analyses were performed using one-way analysis of variance (ANOVA) followed by Sidak’s post hoc test for multiple comparisons. Non-normally distributed data were analyzed using the non-parametric Kruskal–Wallis H test, with Dunn’s post hoc test applied for subsequent pairwise comparisons. Data distribution guided the choice of descriptive statistics and hypothesis testing. Normally distributed datasets are presented as mean ± SEM, whereas non-normally distributed datasets are reported as median (IQR). All statistical tests were two-tailed, and a *p*-value of less than 0.05 was considered statistically significant. Statistical analyses were performed using IBM SPSS Statistics version 30 (GraphPad Software. Inc., San Diego, CA, USA, www.graphpad.com) and GraphPad Prism version 6.5 for Windows (GraphPad Software. Inc., San Diego, CA, USA, www.graphpad.com).

## 3. Results

### 3.1. Reduced Oxygen Saturation Under Hypoxia Disrupts Renal Function and Electrolyte Homeostasis

Blood gas analysis demonstrated that both oxygen concentration and oxygen saturation were significantly lower in the H7 and H14 groups than in their corresponding normoxic groups (Table 1). In contrast, oxygen concentration and saturation in the H7R7 group were markedly higher than those in the H14 group and were not significantly different from those observed in the N14 group (Table 1). In comparison, carbon dioxide concentration did not differ significantly among the P7 or P14 relative groups (Table 1). Blood biochemical analysis further revealed that blood pH values were not significantly altered under different oxygen exposure conditions (Table 1). To further evaluate renal injury induced by hypoxia or hypoxia/reoxygenation, renal function parameters and major serum cations were analyzed. The renal injury marker creatinine was significantly elevated in rats maintained under hypoxic conditions (Table 1). No significant difference in creatinine levels was observed between the H7N7 groups and the H14 group (Table 1). In addition, serum levels of major renal metabolic cations were markedly increased in hypoxia groups after 14 days compared with normoxia groups (Table 1). Compared with the H14 group, cation concentrations were significantly reduced in the H7N7 group (Table 1).

### 3.2. Hypoxia Increases Renal Injury in Neonatal Rats, Partially Reversed by Normoxia

**Figure 1:**
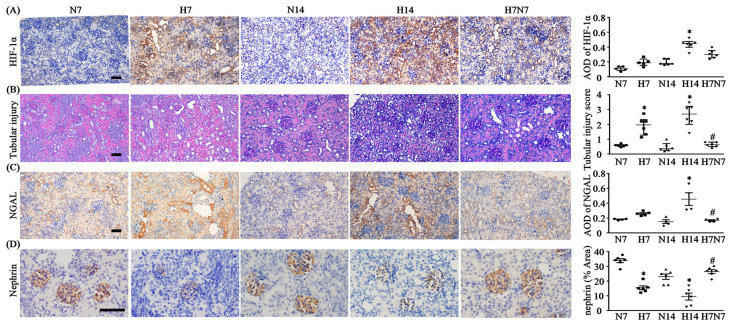
Renal injury and associated biomarkers in the renal cortex of a postnatal hypoxia rat model. (**A**) Expression of the hypoxia marker HIF-1α in the renal cortex, quantified by average optical density (AOD), with HIF-1α-positive staining shown as brown coloration in IHC sections. Representative images were obtained at 200× magnification; scale bar = 50 μm. (**B**) Periodic acid–Schiff (PAS) staining of the renal cortex for the assessment of tubular injury scores, followed by quantitative analysis. Images were acquired at 200× magnification; scale bar = 50 μm. (**C**) Expression of the renal injury marker NGAL in the renal cortex, quantified by AOD, with NGAL-positive staining shown as brown coloration in IHC sections. Images were obtained at 200× magnification; scale bar = 50 μm. (**D**) Glomerular expression of nephrin in each group was quantified as the proportion of nephrin-positive area within the glomerulus, with nephrin staining shown as brown coloration in IHC sections. Images were obtained at 400× magnification; scale bar = 50 μm. For all panels, *n* = 6 per group. Data distribution was assessed using the Shapiro–Wilk normality test to determine whether parametric or non-parametric statistical analyses were applied. Parameters were analyzed using one-way ANOVA followed by the Sidak post hoc test, and are presented as mean ± SEM (**C**,**D**). Parameters were analyzed using the Kruskal–Wallis H test followed by Dunn’s post hoc test correction, and are presented as median (IQR) (**A**,**B**). * *p* < 0.05 versus normoxia groups (N7 and N14); ^#^ *p* < 0.05 versus H14. In the statistical figures, circles represent the N7 group, squares represent the H7 group, triangles represent the N14 group, inverted triangles represent the H14 group, and diamonds represent the H7N7 group. H7: neonatal rats exposed to hypoxia for 7 days after birth; H14: neonatal rats exposed to hypoxia for 14 days after birth; H7N7: neonatal rats exposed to hypoxia for 7 days followed by normoxia for 7 days; N7: neonatal rats maintained under normoxia for 7 days; N14: neonatal rats maintained under normoxia for 14 days; HIF-1α: hypoxia-inducible factor-1α; NGAL: neutrophil gelatinase-associated lipocalin.

Based on the blood biochemical findings, we further investigated renal histopathological changes and injury marker expression. Immunohistochemical analysis showed that HIF-1α expression in the renal cortex was significantly increased in both H7 and H14 groups compared with their respective normoxic controls (Figure 1A). Following 7 days of hypoxia followed by 7 days of normoxia, HIF-1α expression was significantly lower than that in the H14 group but remained significantly higher than that in the N14 group (Figure 1A). PAS staining revealed that tubulointerstitial injury scores (TSI) in the renal cortex were significantly elevated in both H7 and H14 groups compared with normoxic groups (Figure 1B). Renal cortical injury in the H7N7 group was markedly attenuated relative to the H14 group and did not differ significantly from the N14 group (Figure 1B). Consistently, the expression pattern of NGAL, an acute renal cortical injury marker, paralleled the changes observed in TSI (Figure 1C). At the glomerular level, nephrin expression, a key filtration barrier protein, was significantly reduced in hypoxia groups compared with corresponding normoxia groups (Figure 1D). Notably, nephrin expression in the H7N7 group was restored to levels comparable to those in the N14 group (Figure 1D). Analysis of GSI from PAS-stained kidney sections showed that glomerulus injuries were significantly more severe in the H14 group than in the N14 group, whereas this injury was alleviated in the H7N7 group (Supplementary Figure S1).

### 3.3. Hypoxia Induces Fibrotic Remodeling with Incomplete Recovery After Reoxygenation

**Figure 2:**
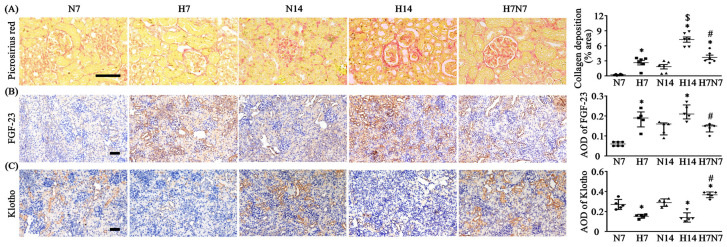
Renal cortical fibrosis and its modulation following reoxygenation in a postnatal hypoxia rat model. (**A**) Picro-Sirius Red staining of rat kidneys to visualize collagen deposition, used to evaluate the degree of renal cortical fibrosis, with fibrotic areas appearing as red coloration in the stained sections. Representative images were obtained at 400× magnification. (**B**) Expression of the fibrosis-associated biomarker FGF-23 in the renal cortex, analyzed at 200× magnification and quantified by average optical density (AOD) within the field, with FGF-23-positive staining shown as brown coloration in IHC sections. (**C**) Expression of the anti-fibrotic biomarker Klotho in the renal cortex, analyzed at 200× magnification and quantified by AOD within the field, with klotho-positive staining shown as brown coloration in IHC sections. For all panels, *n* = 6 per group. Data distribution was assessed using the Shapiro–Wilk normality test to determine whether parametric or non-parametric statistical analyses were applied. Parameters were analyzed using one-way ANOVA followed by the Sidak post hoc test, and are presented as mean ± SEM (**A**,**C**). Parameters were analyzed using the Kruskal–Wallis H test followed by Dunn’s post hoc test correction, and are presented as median (IQR) (**B**) * *p* < 0.05 versus normoxia groups (N7 and N14); ^#^ *p* < 0.05 versus H14 (H7N7 vs. H14); ^$^ *p* < 0.05 versus H14 (H7 vs. H14). In the statistical figures, circles represent the N7 group, squares represent the H7 group, triangles represent the N14 group, inverted triangles represent the H14 group, and diamonds represent the H7N7 group. H7: neonatal rats exposed to hypoxia for 7 days after birth; H14: neonatal rats exposed to hypoxia for 14 days after birth; H7N7: neonatal rats exposed to hypoxia for 7 days followed by normoxia for 7 days; N7: neonatal rats maintained under normoxia for 7 days; N14: neonatal rats maintained under normoxia for 14 days; FGF-23: fibroblast growth factor 23.

PSR staining demonstrated that fibrotic areas in the renal cortex were significantly increased in both the H7 and H14 hypoxia groups compared with normoxia controls (Figure 2A), with a significantly greater extent observed in the H14 group than in the H7 group (Figure 2A). Fibrosis was markedly reduced in the H7N7 group compared with the H14 group (Figure 2A). Regarding the changes in FGF-23 expression in the renal cortex, both the H7 and H14 groups showed a marked increase compared with their corresponding normoxic control groups (Figure 2B). In contrast, the H7N7 group exhibited a decreasing trend in cortical FGF-23 expression compared with the H14 group (Figure 2B). Moreover, the expression of the anti-fibrotic marker klotho was significantly decreased in hypoxia groups relative to normoxia groups (Figure 2C). Following reoxygenation, klotho expression was significantly increased in the H7N7 group compared with both H14 and N14 groups (Figure 2C).

### 3.4. Postnatal Hypoxia Induces Dynamic Iron Accumulation and Ferroptosis, Partially Attenuated by Reoxygenation

**Figure 3:**
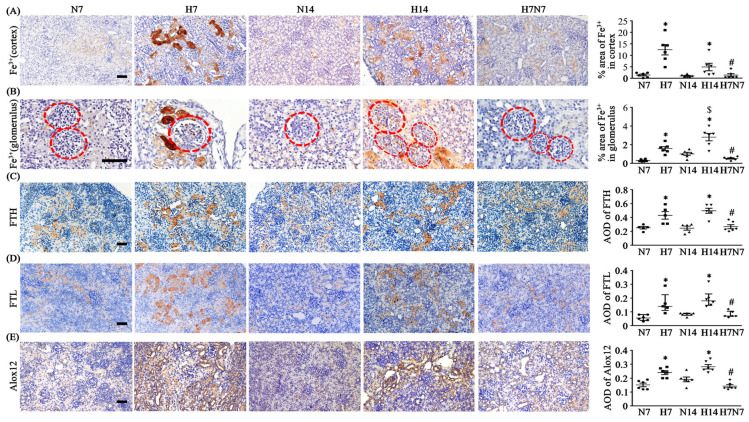
Iron accumulation and ferroptosis-related protein expression in the renal cortex during postnatal hypoxia and reoxygenation. (**A**,**B**) Comparison of iron deposition in renal tubules (**A**) and glomeruli (**B**) (indicated by red dotted circles) at P7 and P14 under postnatal hypoxia. Iron accumulation was detected by Prussian blue staining, with iron deposition shown as brown-stained areas in the sections. Representative images were obtained at 200× magnification for renal tubules and 400× magnification for glomeruli. Scale bar = 50 μm. (**C**,**D**) Expression of FtH (**C**) and FtL (**D**) in renal tubular cells of the cortex, observed at 200× magnification. Scale bar = 50 μm, with FtH- and FtL-positive staining shown as brown coloration in IHC sections. (**E**) Expression of the iron-dependent lipid peroxidation enzyme ALOX12 in the renal cortex of the postnatal hypoxia rat model, observed at 200× magnification. Scale bar = 50 μm, with Alox12-positive staining shown as brown coloration in IHC sections. For all panels, *n* = 6 per group. Data distribution was assessed using the Shapiro–Wilk normality test to determine whether parametric or non-parametric statistical analyses were applied. Parameters were analyzed using one-way ANOVA followed by the Sidak post hoc test, and are presented as mean ± SEM (**B**,**C**,**E**). Parameters were analyzed using the Kruskal–Wallis H test followed by Dunn’s post hoc test correction, and are presented as median (IQR) (**A**,**D**). * *p* < 0.05 versus normoxia groups (N7 and N14); ^#^ *p* < 0.05 versus H14 (H7N7 vs. H14); ^$^ *p* < 0.05 versus H14 (H7 vs. H14). In the statistical figures, circles represent the N7 group, squares represent the H7 group, triangles represent the N14 group, inverted triangles represent the H14 group, and diamonds represent the H7N7 group. ALOX12: arachidonate 12-lipoxygenase; FtH: ferritin heavy chain; FtL: ferritin light chain; H7: neonatal rats exposed to hypoxia for 7 days after birth; H14: neonatal rats exposed to hypoxia for 14 days after birth; H7N7: neonatal rats exposed to hypoxia for 7 days followed by normoxia for 7 days; N7: neonatal rats maintained under normoxia for 7 days; N14: neonatal rats maintained under normoxia for 14 days.

Ferric iron accumulation in the renal cortex and glomeruli was significantly increased in hypoxia groups compared with normoxia groups (Figure 3A,B). Reoxygenation at P7 significantly reduced iron accumulation relative to the H14 group (Figure 3A,B). Within the H14 group, ferric iron levels in the renal cortex were markedly decreased (Figure 3A). The expression levels of FtH and FtL chains were significantly elevated in the renal cortex of hypoxia groups but were markedly reduced in the H7N7 group, returning to levels comparable to those of the N14 group (Figure 3C,D). To assess lipid peroxidation associated with ferroptosis, the expression of Alox12 was examined. Alox12 expression in the renal cortex was significantly increased in both H7 and H14 groups compared with normoxic controls. Notably, Alox12 expression was significantly reduced in the H7N7 group compared with the H14 group and was also lower than that observed in the N14 group (Figure 3E).

## 4. Discussion

Our findings suggest that ferroptosis may be involved in the progression of AKI in postnatal hypoxic rat pups, and that both renal injury and ferroptosis-related alterations were partially attenuated following restoration to normoxic conditions. Our previous study showed that neonatal rats developed significant renal injury and fibrosis after 7 days of postnatal hypoxia [8], and these findings were consistently reproduced in the present study. Histopathological and biochemical assessments revealed comparable patterns of hypoxia-induced renal injury in both P7 and P14 rats maintained under 13% oxygen conditions. Notably, in the P14 model, animals subjected to hypoxia followed by reoxygenation (H7N7 group) exhibited marked recovery of renal injury, accompanied by normalization of ferroptosis- and fibrosis-associated pathways.

The change in arterial oxygen saturation (SaO_2_) during the perinatal period is a critical physiological factor influencing neonatal development and overall health. Clinically, SaO_2_ increases from approximately 65% to 95% after delivery during the early postnatal transition [22]. However, various conditions, including prematurity, congenital heart disease, infection, and other cardiopulmonary dysfunctions, may impair oxygenation and reduce arterial oxygen content. Postnatal hypoxia can result in severe and often irreversible injury to the neonatal brain and other organs, leading to conditions such as cerebral palsy, developmental delay, epilepsy, and multi-organ dysfunction [23]. At the cellular level, hypoxia initiates a cascade of deleterious events, including cell death, neuroinflammation, and long-term neurocognitive impairment [24].

The kidneys, as organs with high oxygen demand, are particularly susceptible to oxygen deprivation. Clinically, ischemia-induced hypoxia is a common cause of both acute kidney injury (AKI) and chronic kidney disease (CKD) [25,26,27]. Oxygen deficiency resulting from ischemia promotes the accumulation of reactive oxygen species (ROS) in renal tubular epithelial cells, thereby triggering acute tubular necrosis. Previous studies have demonstrated that sustained renal hypoxia is a critical driving factor in the transition from AKI to CKD [28]. Although hypoxia is widely recognized as a major risk factor for AKI, investigations focusing on hypoxia-induced AKI in neonates remain relatively limited compared with those in adults. Furthermore, while clinical evidence suggests a strong association between perinatal hypoxia and the development of AKI in neonates, the underlying mechanisms linking postnatal hypoxia to renal injury have yet to be fully elucidated.

In the present study, our rodent model successfully recapitulated the clinical condition of postnatal hypoxia in neonates, as evidenced by reduced arterial oxygen pressure and oxygen saturation (SaO_2_) (Table 1). Concurrently, biochemical parameters indicative of renal injury were significantly elevated under hypoxic conditions (Table 1), supporting the notion that postnatal hypoxia is associated with acute renal dysfunction in neonatal rats. Notably, compared with the H14 group, both oxygenation status and renal injury parameters were markedly improved in the H7N7 group, suggesting a potentially reversible component of hypoxia-induced renal damage following reoxygenation (Table 1).

Histological analysis further demonstrated that the expression of HIF-1α was significantly increased in the renal cortex of hypoxic groups compared with normoxic controls (Figure 1A), indicating that reduced SaO_2_ promotes the stabilization and accumulation of HIF-1α as a key transcription factor regulating adaptive responses to hypoxia, including erythropoiesis, inflammation, and antioxidative pathways [29]. Consistently, semiquantitative analysis of the tubular injury score (TSI) revealed a significant increase in hypoxic groups relative to their corresponding normoxic controls, indicating that postnatal hypoxia induces substantial tubulointerstitial damage (Figure 1B). NGAL, a well-established early biomarker of tubular injury [30], has also been implicated in the progression of renal fibrosis through modulation of matrix metalloproteinase-9 (MMP-9) degradation [31]. In our study, the upregulation of NGAL in the renal cortex of hypoxic rat pups, together with the parallel increase in TSI, supports the presence of tubular injury and confirms that reduced SaO_2_ effectively induces renal damage in this model (Figure 1C).

In addition to tubular injury, glomerular damage was evaluated based on morphological features and nephrin expression [32]. Both semiquantitative glomerular injury scores (GSI) and nephrin expression levels indicated significantly increased glomerular injury in postnatal hypoxic pups (Figure 1D and Supplementary Figure S1). Due to the immature and atypical glomerular morphology observed in P7 rats, GSI analysis was limited to P14 animals (Supplementary Figure S1). Importantly, both histological alterations and molecular markers of renal injury were significantly ameliorated in the H7N7 group compared with the H14 group. Collectively, these findings demonstrate that our postnatal hypoxia model reliably reproduces renal injury observed in neonates with systemic hypoxia. Furthermore, the observed improvement following reoxygenation indicates that hypoxia-induced renal injury is, at least in part, oxygen-dependent and potentially reversible upon restoration of normal oxygen availability.

Progressive renal fibrosis and increased expression of pro-fibrotic markers are hallmark features of the transition from AKI to chronic kidney disease (CKD). A previous long-term clinical cohort study demonstrated that approximately 10% of pediatric patients with AKI admitted to the pediatric intensive care unit (PICU) progressed to varying stages of CKD during a three-year follow-up period [33]. Experimental studies have further shown that most rodent models of AKI, regardless of the induction method, exhibit the potential to progress toward CKD through sustained fibrotic remodeling. In the present study, the H7 and H14 groups exhibited significantly greater fibrotic changes compared with their corresponding normoxic controls (N7 and N14), as evidenced by increased collagen deposition, elevated FGF-23 expression, and decreased klotho expression in the tubulointerstitial regions of the renal cortex (Figure 2). Notably, these fibrosis-related parameters displayed distinct patterns in the H7N7 group following reoxygenation. Although collagen deposition in the H7N7 group remained higher than that in the N14 group, despite partial attenuation, FGF-23 expression returned to levels comparable to those observed in the N14 group (Figure 2A,B). In contrast, klotho expression was markedly upregulated in the H7N7 group, even exceeding that of the N14 group (Figure 2C). These findings suggest that restoration of oxygen supply may partially mitigate the progression of renal fibrosis; however, extracellular matrix accumulation initiated during early hypoxic injury may not be fully reversible.

The persistent elevation of collagen deposition in the H7N7 group indicates that early postnatal hypoxic insult may trigger fibrotic remodeling processes that continue despite subsequent normalization of oxygen tension. Meanwhile, the normalization of FGF-23 expression following reoxygenation implies that hypoxia-induced dysregulation of mineral metabolism–related signaling may be reversible upon restoration of systemic oxygenation. Interestingly, the pronounced upregulation of klotho in the H7N7 group may represent a compensatory response to prior hypoxic stress. Given that klotho has been reported to negatively regulate FGF-23 signaling and exert renoprotective effects against fibrotic progression [34], its increased expression in the reoxygenation group may contribute to the suppression of FGF-23 levels and potentially limit further fibrotic deterioration.

It is well established that ferroptotic pathways play a critical role in the pathogenesis of AKI. Even prior to the formal definition of ferroptosis by Dixon et al. in 2012, both clinical and experimental studies had reported that iron chelators conferred protective effects against AKI, thereby implicating iron-dependent oxidative injury as a key contributor to renal damage [35]. Subsequent studies further demonstrated that various animal models of AKI exhibit dysregulated iron metabolism and respond to iron chelation therapy [36,37,38]. More recently, accumulating evidence has suggested that ferroptosis is not only involved in the acute phase of kidney injury but also plays an important role in the progression from AKI to CKD [39,40]. In the context of postnatal hypoxia, disruption of iron homeostasis may represent a crucial mechanistic link between hypoxic stress and ferroptotic injury in renal tubular epithelial cells. Increasing evidence indicates that ferroptosis and renal fibrosis share overlapping molecular pathways during the AKI-to-CKD transition. For instance, epithelial–mesenchymal transition (EMT) during renal fibrogenesis has been reported to activate ferritinophagy, resulting in the degradation of ferritin heavy chain (FtH) and the subsequent release of substantial amounts of labile iron into tubular epithelial cells, thereby promoting ferroptotic cell death [40]. Consistent with these findings, our results demonstrated that both iron accumulation and FtH expression were markedly increased in the H7 and H14 groups compared with the N7 and N14 groups (Figure 3A,C), supporting the involvement of ferroptosis-related iron dysregulation in postnatal hypoxia-induced renal injury. The concurrent increase in ferritin and ferric iron deposition further suggests the presence of persistent iron overload in renal tubular epithelial cells under hypoxic conditions.

Interestingly, ferric iron accumulation in the H14 group was significantly lower than that in the H7 group (Figure 3A), which may reflect a temporal modulation of ferroptosis during the later stages of hypoxic exposure. Importantly, the sustained upregulation of the iron-dependent lipid peroxidation enzyme ALOX12 further implies that ferroptosis-related pathways remain functionally relevant despite increased ferritin expression. These findings suggest that ferritin upregulation may represent a compensatory response to excess intracellular iron, aiming to sequester labile iron and mitigate ferroptotic damage, rather than reflecting changes in iron distribution.

Notably, HIF-1α, a key regulator under hypoxic conditions, has been reported to attenuate ferroptosis through post-translational mechanisms, including upregulation of FtH and suppression of ferritinophagy, thereby limiting iron release and lipid peroxidation [41,42]. In line with these findings, our data demonstrated that FtH expression remained elevated at both H7 and H14 stages, whereas the extent of detectable free iron deposition was reduced at H14 compared to H7. These results support a temporally regulated ferroptotic response in postnatal hypoxia-induced kidney injury. Specifically, early-stage hypoxia (H7) appears to be associated with increased ferroptosis-related activity, whereas prolonged hypoxia (H14) may be associated with a shift toward a ferroptosis-suppressed but iron-loaded state. This transition may reflect a potential adaptive response possibly associated with HIF-1α signaling, which limits excessive ferroptotic damage while allowing persistent iron accumulation. Furthermore, this temporal shift may contribute to the progression from acute injury to fibrotic remodeling, as ferroptosis becomes less dominant and fibrosis-related pathways take precedence. Nevertheless, the observation that ferroptosis-related proteins and iron levels were restored toward baseline following normoxic recovery (H7N7 group) suggests that ferroptosis remains functionally relevant, even at later stages, and may still contribute to renal injury dynamics.

Several limitations should be acknowledged. First, ferroptosis involvement was inferred from indirect markers (iron accumulation, ALOX12, and ferritin dynamics) without ferroptosis-specific interventions to establish causality. Second, ferritinophagy, iron flux, and downstream HIF-1α–related pathways were not directly assessed. Finally, the mechanisms underlying the partial recovery in the H7N7 group and the persistence of fibrosis remain unclear and warrant further investigation.

## 5. Conclusions

In conclusion, our findings demonstrate that ferroptosis may contribute to the pathophysiology of postnatal hypoxia-induced AKI and is closely associated with alterations in iron handling within renal tubular cells. The observed reversal of ferroptosis-related changes following normoxic recovery highlights the potential therapeutic relevance of targeting iron metabolism and ferroptotic pathways. Modulation of ferroptosis may therefore represent a potential therapeutic strategy for mitigating hypoxia-related renal injury and preventing AKI-to-CKD progression.

## Figures and Tables

**Table 1:** Whole-blood biochemical analysis in neonatal rats subjected to postnatal hypoxia. Whole-blood samples were collected without centrifugation immediately after decapitation, and analyzed for blood gas parameters, acid-base status, and electrolyte concentrations.

Parameter/Group	N7	H7	N14	H14	H7N7
pO_2_ ^b^ (mmHg)	54.00 (49.85–58.15)	21.35 * (16.18–28.60)	48.50 (12.25–51.13)	28.75 * (22.78–31.53)	45.65 ^#^ (38.00–52.15)
pCO_2_ ^b^ (mmHg)	48.95 (48.30–50.95)	48.95 (46.94–55.18)	48.95 (47.30–50.35)	52.40 (40.48–53.5)	50.15 (49.28–52.15)
cSO_2_ ^a^ (%)	82.15 ± 5.91	41.13 ±14.48 *	81.78 ± 1.94	58.26 ± 1.94 *	79.50 ± 3.42 ^#^
cTCO_2_^a^ (mmol/L)	30.88 ± 0.39	29.45 ± 1.11 *	27.15 ± 0.19	25.59 ± 0.19 *	28.48 ± 0.18 ^#^
pH ^a^	7.41 ± 0.02	7.40 ± 0.06	7.39 ± 0.01	7.40 ± 0.00	7.39 ± 0.06
Creatinine ^b^ (mg/dL)	0.39 (0.36–0.42)	0.47 * (0.42–0.62)	0.41 (0.36–0.45)	0.51 * (0.45–0.65)	0.52 * (0.48–0.52)
Sodium ^b^ (mmol/L)	126.00 (125.00–127.00)	125.00 (123.20–128.00)	126.00 (125.00–127.00)	128.00 * (127.00–128.75)	126.50 ^#^ (126.00–127.00)
Potassium ^b^ (mmol/L)	5.90 (5.40–6.20)	8.90 * (7.54–9.70)	7.50 (7.25–7.50)	8.25 (7.55–8.73)	7.40 ^#^ (7.03–7.50)
Chloride ^b^ (mmol/L)	98.00 (96.00–98.00)	102.60 * (102.00–103.00)	99.00 (98.00–7.50)	103.00 * (102.25–103.75)	100.50 ^#^ (100.00–102.00)
Calcium ^a^ (mmol/L)	1.37 ± 0.02	1.36 ± 0.06	1.40 ± 0.03	1.45 ± 0.02	1.37 ± 0.06 ^#^

^a^. The values are presented as the means ± SEM, *p* values were estimated by one-way ANOVA followed by the Sidak post hoc test. ^b^. The values are presented as the median (IQR), *p* values were estimated by Kruskal–Wallis H test followed by Dunn’s post hoc test. * *p* < 0.05 versus normoxia groups (N7 and N14, *n* = 6–8). ^#^ *p* < 0.05 versus H14 (*n* = 8). H7: neonatal rats exposed to hypoxia for 7 days after birth; H14: neonatal rats exposed to hypoxia for 14 days after birth; N7: neonatal rats maintained under normoxia for 7 days; N14: neonatal rats maintained under normoxia for 14 days; H7N7: neonatal rats exposed to hypoxia for 7 days followed by normoxia for 7 days. pCO_2_: partial pressure of carbon dioxide; pO_2_: partial pressure of oxygen; cTCO_2_: total carbon dioxide concentration; cSO_2_: oxygen saturation.

## Data Availability

The original contributions presented in this study are included in the article. Further inquiries can be directed to the corresponding author.

## Supplementary Materials

The following supporting information can be downloaded at: https://www.mdpi.com/article/10.3390/antiox15050582/s1, Figure S1: Histopathological evaluation and semiquantitative analysis of glomerular injury in P14 rats across experimental groups.

## Abbreviations

The following abbreviations are used in this manuscript:

ACSL4	Acyl-CoA synthetase long-chain family member 4
AKI	Acute kidney injury
ALOX12	Arachidonate 12-lipoxygenase
CKD	Chronic kidney disease
DAB	3,3′-diaminobenzidine
EDTA	Ethylenediaminetetraacetic acid
EMT	Epithelial–mesenchymal transition
FGF23	Fibroblast growth factor 23
GPX4	Glutathione peroxidase 4
H14	Hypoxia group at postnatal day 14
H7	Hypoxia group at postnatal day 7
H7N7	Hypoxia for 7 days followed by 7 days of normoxia (reoxygenation)
HIF-1α	Hypoxia-inducible factor-1 alpha
IHC	Immunohistochemistry
LP-OOH	Lipid hydroperoxide
MMP-9	Matrix metalloproteinase-9
N14	Normoxia group at postnatal day 14
N7	Normoxia group at postnatal day 7
NGAL	Neutrophil gelatinase-associated lipocalin
OSOM	Outer stripe of outer medulla
P0	Postnatal day 0
P14	Postnatal day 14
P7	Postnatal day 7
PAS	Periodic acid-Schiff
PSR	Picrosirius red
ROS	Reactive oxygen species

## References

[1] Kingdom J.C., Kaufmann P. (1997). Oxygen and placental villous development: Origins of fetal hypoxia. Placenta.

[2] Mouradian G.C., Lakshminrusimha S., Konduri G.G. (2021). Perinatal Hypoxemia and Oxygen Sensing. Compr. Physiol..

[3] Romanowicz J., Guerrelli D., Dhari Z., Mulvany C., Reilly M., Swift L., Vasandani N., Ramadan M., Leatherbury L., Ishibashi N. (2021). Chronic perinatal hypoxia delays cardiac maturation in a mouse model for cyanotic congenital heart disease. Am. J. Physiol. Heart Circ. Physiol..

[4] Farahani R., Kanaan A., Gavrialov O., Brunnert S., Douglas R.M., Morcillo P., Haddad G.G. (2008). Differential effects of chronic intermittent and chronic constant hypoxia on postnatal growth and development. Pediatr. Pulmonol..

[5] Plotnikov E.Y., Pavlenko T.A., Pevzner I.B., Zorova L.D., Manskikh V.N., Silachev D.N., Sukhikh G.T., Zorov D.B. (2017). The role of oxidative stress in acute renal injury of newborn rats exposed to hypoxia and endotoxin. FEBS J..

[6] Sweetman D.U., Riordan M., Molloy E.J. (2013). Management of renal dysfunction following term perinatal hypoxia-ischaemia. Acta Paediatr..

[7] Duzova A., Bakkaloglu A., Kalyoncu M., Poyrazoglu H., Delibas A., Ozkaya O., Peru H., Alpay H., Soylemezoglu O., Gur-Guven A. (2010). Etiology and outcome of acute kidney injury in children. Pediatr. Nephrol..

[8] Chu Y.T., Chen B.H., Chen H.H., Lee J.C., Kuo T.J., Chiu H.C., Lu W.H. (2023). Hypoxia-Induced Kidney Injury in Newborn Rats. Toxics.

[9] Dixon S.J., Lemberg K.M., Lamprecht M.R., Skouta R., Zaitsev E.M., Gleason C.E., Patel D.N., Bauer A.J., Cantley A.M., Yang W.S. (2012). Ferroptosis: An iron-dependent form of nonapoptotic cell death. Cell.

[10] Broxmeyer H.E., Cooper S., Levi S., Arosio P. (1991). Mutated recombinant human heavy-chain ferritins and myelosuppression in vitro and in vivo: A link between ferritin ferroxidase activity and biological function. Proc. Natl. Acad. Sci. USA.

[11] Zhou Q., Li T., Qin Q., Huang X., Wang Y. (2022). Ferroptosis in lymphoma: Emerging mechanisms and a novel therapeutic approach. Front. Genet..

[12] Chen X., Yu C., Kang R., Tang D. (2020). Iron Metabolism in Ferroptosis. Front. Cell Dev. Biol..

[13] Feng H., Stockwell B.R. (2018). Unsolved mysteries: How does lipid peroxidation cause ferroptosis?. PLoS Biol..

[14] Li J., Cao F., Yin H.L., Huang Z.J., Lin Z.T., Mao N., Sun B., Wang G. (2020). Ferroptosis: Past, present and future. Cell Death Dis..

[15] Li C., Wu Z., Xue H., Gao Q., Zhang Y., Wang C., Zhao P. (2022). Ferroptosis contributes to hypoxic-ischemic brain injury in neonatal rats: Role of the SIRT1/Nrf2/GPx4 signaling pathway. CNS Neurosci. Ther..

[16] Liu X.Q., Shi M.Z., Bai Y.T., Su X.L., Liu Y.M., Wu J.C., Chen L.R. (2024). Hypoxia and ferroptosis. Cell. Signal.

[17] Peeples E.S., Genaro-Mattos T.C. (2022). Ferroptosis: A Promising Therapeutic Target for Neonatal Hypoxic-Ischemic Brain Injury. Int. J. Mol. Sci..

[18] Zhang L., Luo F., Yuan N., Yin J., Shen B., Chai Y., Sun L., Wang X., Yin L., Luo C. (2025). Research progress of ferroptosis in acute kidney injury. Front. Cell Dev. Biol..

[19] Feng Q., Yu X., Qiao Y., Pan S., Wang R., Zheng B., Wang H., Ren K.D., Liu H., Yang Y. (2022). Ferroptosis and Acute Kidney Injury (AKI): Molecular Mechanisms and Therapeutic Potentials. Front. Pharmacol..

[20] Wu C.J., Li Y.H., Wu F.Z., Chen H.H. (2024). Eplerenone improves hyperglycemia and sympathetic excitation in chronic renocardiac syndrome in rats. Naunyn Schmiedebergs Arch. Pharmacol..

[21] Moos T., Mollgard K. (1993). A sensitive post-DAB enhancement technique for demonstration of iron in the central nervous system. Histochemistry.

[22] Lee H.C., Strand M.L., Finan E., Illuzzi J., Kamath-Rayne B.D., Kapadia V., Mahgoub M., Niermeyer S., Schexnayder S.M., Schmolzer G.M. (2025). Part 5: Neonatal Resuscitation: 2025 American Heart Association and American Academy of Pediatrics Guidelines for Cardiopulmonary Resuscitation and Emergency Cardiovascular Care. Circulation.

[23] Pedroza-Garcia K.A., Calderon-Vallejo D., Quintanar J.L. (2022). Neonatal Hypoxic-Ischemic Encephalopathy: Perspectives of Neuroprotective and Neuroregenerative Treatments. Neuropediatrics.

[24] Li F., Wang L., Li J.W., Gong M., He L., Feng R., Dai Z., Li S.Q. (2011). Hypoxia induced amoeboid microglial cell activation in postnatal rat brain is mediated by ATP receptor P2X4. BMC Neurosci..

[25] Ow C.P.C., Ngo J.P., Ullah M.M., Hilliard L.M., Evans R.G. (2018). Renal hypoxia in kidney disease: Cause or consequence?. Acta Physiol..

[26] Shu S., Wang Y., Zheng M., Liu Z., Cai J., Tang C., Dong Z. (2019). Hypoxia and Hypoxia-Inducible Factors in Kidney Injury and Repair. Cells.

[27] Eckardt K.U., Bernhardt W.M., Weidemann A., Warnecke C., Rosenberger C., Wiesener M.S., Willam C. (2005). Role of hypoxia in the pathogenesis of renal disease. Kidney Int. Suppl..

[28] Tanaka S., Tanaka T., Nangaku M. (2014). Hypoxia as a key player in the AKI-to-CKD transition. Am. J. Physiol. Ren. Physiol..

[29] Li Q.Y., Liu F., Tang X., Fu H., Mao J. (2022). Renoprotective Role of Hypoxia-Inducible Factors and the Mechanism. Kidney Dis..

[30] Bolignano D., Donato V., Coppolino G., Campo S., Buemi A., Lacquaniti A., Buemi M. (2008). Neutrophil gelatinase-associated lipocalin (NGAL) as a marker of kidney damage. Am. J. Kidney Dis..

[31] Tan R.J., Liu Y. (2012). Matrix metalloproteinases in kidney homeostasis and diseases. Am. J. Physiol. Ren. Physiol..

[32] Chen Y., Lin L., Tao X., Song Y., Cui J., Wan J. (2019). The role of podocyte damage in the etiology of ischemia-reperfusion acute kidney injury and post-injury fibrosis. BMC Nephrol..

[33] Mammen C., Al Abbas A., Skippen P., Nadel H., Levine D., Collet J.P., Matsell D.G. (2012). Long-term risk of CKD in children surviving episodes of acute kidney injury in the intensive care unit: A prospective cohort study. Am. J. Kidney Dis..

[34] Lu X., Hu M.C. (2017). Klotho/FGF23 Axis in Chronic Kidney Disease and Cardiovascular Disease. Kidney Dis..

[35] Sharma S., Leaf D.E. (2019). Iron Chelation as a Potential Therapeutic Strategy for AKI Prevention. J. Am. Soc. Nephrol..

[36] Paller M.S., Hedlund B.E. (1988). Role of iron in postischemic renal injury in the rat. Kidney Int..

[37] Zarjou A., Bolisetty S., Joseph R., Traylor A., Apostolov E.O., Arosio P., Balla J., Verlander J., Darshan D., Kuhn L.C. (2013). Proximal tubule H-ferritin mediates iron trafficking in acute kidney injury. J. Clin. Investig..

[38] Petronilho F., Constantino L., de Souza B., Reinke A., Martins M.R., Fraga C.M., Ritter C., Dal-Pizzol F. (2009). Efficacy of the combination of N-acetylcysteine and desferrioxamine in the prevention and treatment of gentamicin-induced acute renal failure in male Wistar rats. Nephrol. Dial. Transplant..

[39] Gong S., Zhang A., Yao M., Xin W., Guan X., Qin S., Liu Y., Xiong J., Yang K., Xiong L. (2023). REST contributes to AKI-to-CKD transition through inducing ferroptosis in renal tubular epithelial cells. JCI Insight.

[40] Guo R., Duan J., Pan S., Cheng F., Qiao Y., Feng Q., Liu D., Liu Z. (2023). The Road from AKI to CKD: Molecular Mechanisms and Therapeutic Targets of Ferroptosis. Cell Death Dis..

[41] Huang B.W., Miyazawa M., Tsuji Y. (2014). Distinct regulatory mechanisms of the human ferritin gene by hypoxia and hypoxia mimetic cobalt chloride at the transcriptional and post-transcriptional levels. Cell. Signal.

[42] Yanatori I., Nishina S., Kishi F., Hino K. (2023). Newly uncovered biochemical and functional aspects of ferritin. FASEB J..

