# Supplementary material for: Dynamic Regulation of Ferroptosis in a Neonatal Rat Model of Postnatal Hypoxia-Induced Acute Kidney Injury
Source: Antioxidants (Basel). 2026 May 4;15(5):582. doi: 10.3390/antiox15050582 (PMC13203842; doi:10.3390/antiox15050582)
Supplement: Supplementary file 1 [file antioxidants-15-00582-s001.zip › antioxidants-4258778-supplementary.pdf]

1. Supplementary Material and Method

1.1. Glomerulosclerosis index (GSI)

GSI was evaluated based on the area fraction of glomerular abnormalities observed at 400× magnification. Morphological criteria included basement membrane thickening, mesangial extracellular matrix expansion (PAS-positive area), lobulation or atrophy of the capillary tuft, and crescent formation [1]. Glomerular injury severity was graded on a five-point scale as follows: 1) Grade 0: no detectable glomerular abnormality; 2) Grade 1: abnormalities involving <25% of the glomerular area; 3) Grade 2: abnormalities involving 25–50% of the glomerular area; 4) Grade 3: abnormalities involving 50–75% of the glomerular area; and 5) Grade 4: abnormalities involving >75% of the glomerular area. The final GSI for each sample was calculated as the mean score of all evaluated glomeruli within the tissue section.

1.2. *Statistical Analysis*

All results were calculated using a parametric one-way analysis of variance (ANOVA) followed by Sidak's post hoc test for multiple comparisons. All statistical graphs are presented as mean ± standard deviation (mean ± SEM). A p value of <0.05 was considered statistically significant. Statistical analyses were performed using IBM SPSS Statistics Version 20 software (IBM Corp., Armonk, NY, USA, 2011) and GraphPad Prism version 6.5 for Windows (GraphPad Software. Inc., San Diego, CA, USA www.graphpad.com).

2. Supplementary Figure

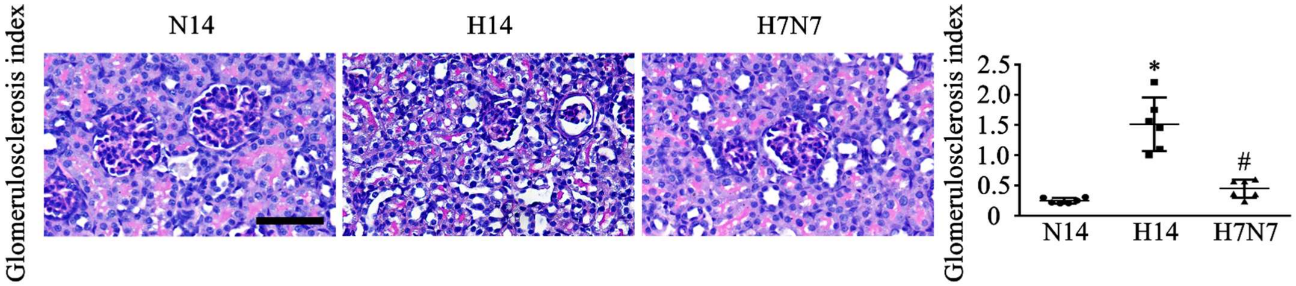

Figure S1. Histopathological evaluation and semiquantitative analysis of glomerular injury in P14 rats across experimental groups.

Renal tissues were stained with PAS and examined at 400× magnification to assess glomerular pathological features, including PAS-positive areas. Glomerular injury was semiquantitatively evaluated using the GSI to compare the extent of glomerular damage among groups. For all panels, $n = 6$ per group. Data are presented as mean ± SEM. Statistical analysis was performed using the one-way analysis of variance (ANOVA) followed by Sidak's post hoc test for multiple comparisons. *P < 0.05 versus N14; #P < 0.05 versus H14. In the statistical figures, circles represent the N14 group, squares represent the H14 group, triangles represent the H7N7 group. GSI: glomerulosclerosis index; H14: neonatal rats exposed to hypoxia for 14 days after birth; H7N7: neonatal rats exposed to hypoxia for 7 days followed by normoxia for 7 days; N14: neonatal rats maintained under normoxia for 14 days; PAS: periodic acid-Schiff staining.

Supplementary References

1. Wu, C.J.; Li, Y.H.; Wu, F.Z.; Chen, H.H. Eplerenone improves hyperglycemia and sympathetic excitation in chronic renocardiac syndrome in rats. *Naunyn Schmiedebergs Arch Pharmacol* **2024**, *397*, 1081-1092, doi:10.1007/s00210-023-02665-5.
